# Supplementary material for: Hot Cordilleran hinterland promoted lower crust mobility and decoupling of Laramide deformation
Source: Nat Commun. 2024 May 4;15:3750. doi: 10.1038/s41467-024-48182-8 (PMC11069518; doi:10.1038/s41467-024-48182-8)
Supplement: Supplementary file 1 — Supplementary Information [file 41467_2024_48182_MOESM1_ESM.pdf]

Supporting Information for

Hot Cordilleran hinterland promoted lower crust mobility and decoupling of Laramide deformation

Dominik R. Vlaha[1,2,3]*, Andrew V. Zuza[1,3], Lin Chen[4], and Matthieu Harlaux[5]

1. *Nevada Bureau of Mines and Geology, University of Nevada, Reno, NV 89557, USA*
2. *Department of Geological Sciences and Engineering, University of Nevada, Reno, NV 89557, USA*
3. *Nevada Geosciences, University of Nevada, Reno, NV 89557, USA*
4. *State Key Laboratory of Lithospheric Evolution, Institute of Geology and Geophysics, Chinese Academy of Sciences, Beijing 100029, China*
5. *BRGM - French Geological Survey, 45060 Orléans, France*

*Corresponding author: Dominik R. Vlaha (dvlaha@unr.edu)

This PDF file includes:

1. Supplementary methods and data compilation
    - Fig. S1. Pre-Cenozoic extension palinspastic reconstructions.
    - Table S1. Parameters for conductive thermal curves

2. Discussion of heating mechanisms and possible slab refrigeration

3. Basal shear analytical solution shows lower-crust decoupling
    - Fig. S2. Hinterland velocity and strain rate distribution.

Supplementary References All citations in the Supplementary Text and in the Dataset are to these Supplementary References, not to the references in the main article or Methods section, which if necessary are repeated here with the appropriate (different) number.

Other supporting materials for this manuscript include the following:
HinterlandTemperatureCompilation_MasterList.xlsx contains physical locations and temperature versus depth estimates for all samples plotted in Fig. 2C. Additionally, raw IFORS data for RSCM temperature estimates and additional remarks are made for the northern Pequop and southern Ruby Mountains.

1. Supplementary methods and data compilation

Timing of peak thermal conditions

Compiled pressure temperature and time (P-T-t) data and argon thermochronology indicate regional temperatures were attained in the Late Cretaceous and persisted to the Paleocene (ca. 90–60 Ma). Here, we compile published geochronology and thermochronology to estimate the relative age of Raman spectroscopy on carbonaceous material (RSCM) thermometry peak temperature estimates. Compiled geochronology consists of U-Pb geochronology on metamorphic zircon, monazite, and titanite, and $^{176}Lu/^{177}Hf$ and $^{147}Sm/^{144}Nd$ garnet geochronology. U-Pb ages on metamorphic zircon, monazite, and titanite are interpreted to record peak regional metamorphic conditions at closure temperatures of >900°C [1,2], ~725°C [3] and ~750°C [4], respectively. Garnet crystallization ages are also interpreted to record the timing of peak metamorphic conditions, with closure temperatures for the $^{176}Lu/^{177}Hf$ and $^{147}Sm/^{144}Nd$ systems at >900 to 1000°C[5,6] and 650 to 700°C[7], respectively.

Argon thermochronology tracks cooling through a specific closure temperature and a combination of different thermochronometers within the sample can record a time-transgressive cooling history. Specifically, the effective closure temperatures of Ar diffusion in biotite, muscovite, and hornblende are 300–350°C, 350–400°C, and 400–600°C, respectively[8–11].

In the Ruby Mountains-East Humboldt Range metamorphic core complex (REH) region, $^{40}Ar/^{39}Ar$ dates from Cambrian samples in the Pequop Mountains, which experienced temperatures >400°C, yielded predominantly 68–78 Ma ages[12]. This observation constrains that the samples cooled from *T* conditions through the closure temperature of argon diffusion in muscovite or biotite, which implies that peak thermal conditions were attained in the Late Cretaceous. Additionally, these samples record Eocene (ca. 42 Ma) potassium feldspar $^{40}Ar/^{39}Ar$ thermochronology ages, which suggest

the structurally deepest samples in the Pequop mountains did not experience Eocene thermal resetting. Late Cretaceous ^{40}Ar/^{39}Ar thermochronology ages are also recorded in the Toano-Goshute mountains and the Pilot Range[13] (Fig. 2A-4). Evidence for voluminous Late Cretaceous two-mica granite generation is, at least in part, by in-situ partial melting during regional metamorphism[14–19]. Leucogranites yielded Late Cretaceous U-Pb ages on zircon and monazite, dated at ca. 69–92 Ma In the central Ruby Mountains[19,20](Fig. 2A-5), ca. 72–78 Ma in the northern Ruby mountains[21], and ca. 72–90 Ma in the East Humboldt Range[22,23](Fig. 2A-2). Metamorphic rims on zircons from Jurassic and Archean intrusions are dated at ca. 70–90 Ma in the REH[18,23–26](Fig. 2A-1). A ^{176}Lu/^{177}Hf garnet age of ca. 83 Ma from the Wood hills[27](Fig. 2A-3) and U-Pb on metamorphic sphene gave a crystallization age of ca. 84 Ma from the northern Pequop mountains[28], providing further evidence for Late Cretaceous heating.

In the broader Snake Range area, peak thermal conditions occurred in the Late Cretaceous[14,15,29]. Late Cretaceous metamorphism is dated at ca. 75 Ma in the Kern Mountains(U-Pb zircon[30]), ca. 80–86 Ma and 160 Ma in the southern Snake Range(Fig. 2A-11, K-Ar muscovite[30–32]), ca. 70–100 Ma in the northern Snake Range(Fig. 2A-9; U-Pb zircon and ^{147}Sm/^{144}Nd monazite[33]; U-Pb zircon[14]; ^{147}Sm/^{144}Nd, ^{176}Lu/^{177}Hf garnet and U-Th-Pb zircon[34]; U-Pb zircon[35]). Additionally, metamorphic cooling ages of ca. 61–90 Ma in the Schell Creek Range (Fig. 2A-7 and 10; K-Ar and ^{40}Ar/^{39}Ar muscovite[14,32]), ca. 73 Ma in the Deep Creek Range (Fig. 2A-6, ^{40}Ar/^{39}Ar hornblende[36]) and ca. 75–78 Ma in the northern Snake Range (Fig. 2A- 8 and 9; U-Pb monazite and ^{40}Ar/^{39}Ar hornblende[37,38]) are well constrained to the Late Cretaceous.

In summary, available evidence constrains that the highest observed temperatures in eastern Nevada were attained in the Late Cretaceous, ca. 90–60 Ma. Later heating was less significant and did not impact the peak temperature results from the RSCM dataset.

Methods for reconstructing paleo-depth of peak-temperature samples

To quantify the pre-extensional paleo-depth of our peak-temperature samples, we drafted and restored slip along major Cenozoic structures across five ranges along the margins of the REH. The cross sections were constrained via geologic mapping in the central Pequop Mountains, along RSCM transects, and published geological maps (from east to west), the Toano-Goshute Mountains[39]; the Pequop Mountains[12,40]; the Spruce Mountains [41,42]; the southern Ruby Mountains[43]; and the regional geology[44]. The regional Paleogene subvolcanic unconformity is not well preserved in the primary mapping area[44], therefore our transects were selected to maximize across-strike exposures of relatively undeformed stratigraphic sections. Stratigraphic thicknesses were determined from geometric constraints along our transects whenever possible. If rock units were not confidently differentiated along the transect, then thicknesses were used from published geologic maps or from isopach maps from ref. [45]. All stratigraphic units were grouped together by geologic period and hanging wall units along range bounding faults were excluded from our cross sections unless there is available drill core data. Cenozoic extensional rotation can be evidenced by tilted sedimentary and volcanic units above the regional Paleogene unconformity[43]. Published tilted Tertiary strata were projected to transects that did not expose the unconformity[46,47].

Paleo-depths were estimated from our restored cross sections beneath the approximate erosional surface and below the reconstructed top of the highest observed unit from each range. For transects that did not expose the Paleogene unconformity, we interpreted the erosional surface from published subcrop maps[48,49] and nearby map relationships. In the northern Pequop Mountains, samples collected from the hanging wall of the Independence thrust were excluded from our paleo-depth estimates due to structural complexities. Instead, we assume samples collected from the Independence

thrust hanging wall experienced peak temperatures at their approximate stratigraphic depths.

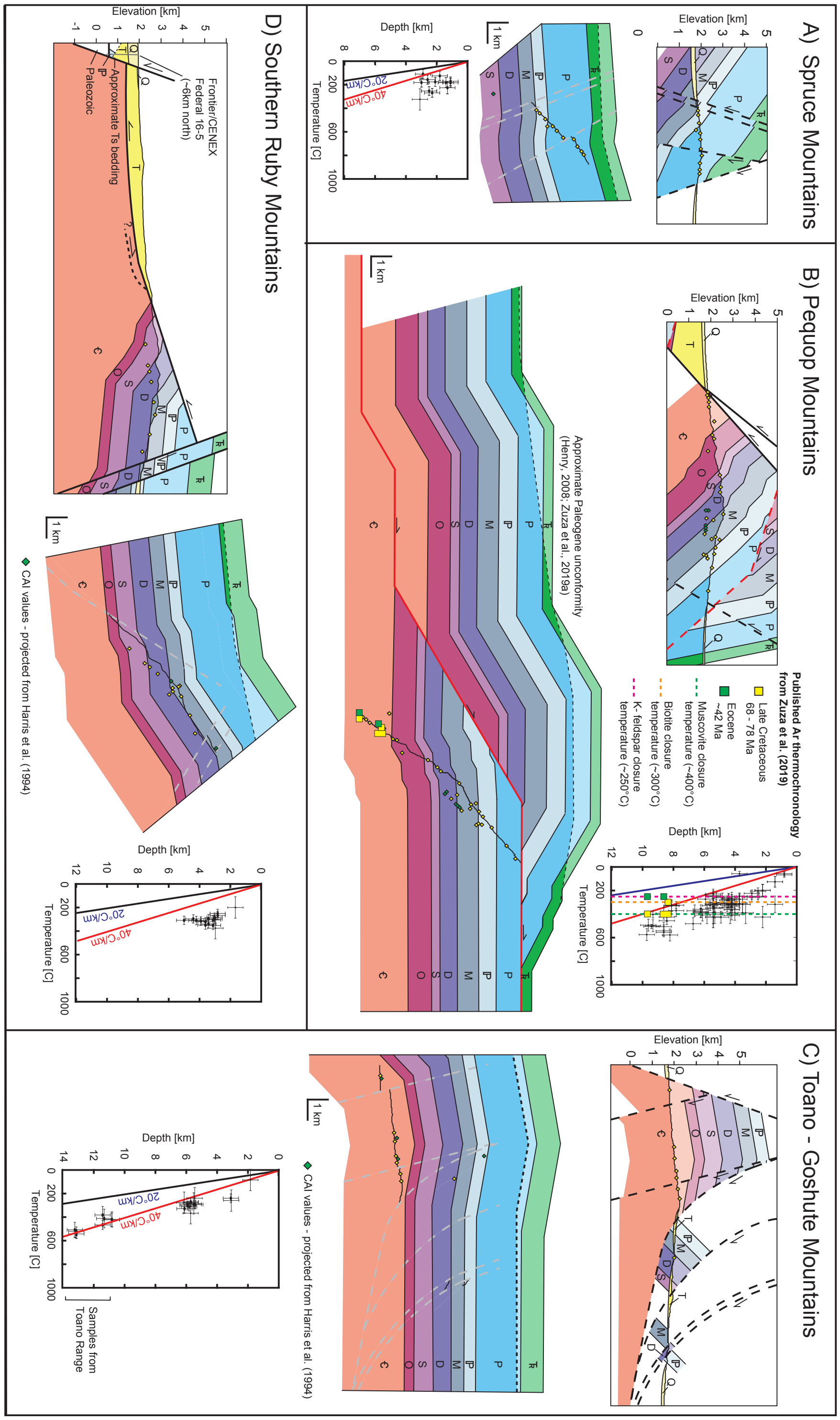

Fig. S1. Pre-Cenozoic extension palinspastic reconstructions. Palinspastic reconstructions restoring slip along major Cenozoic structures and local temperature versus depth data from the (A) Spruce Mountains, (B) Pequop Mountains, (C) Toano-Goshute Mountains, and (D) southern Ruby Mountains. Published argon thermochronology in the Pequop mountains show no Eocene thermal resetting, indicating the structurally deepest samples attained peak temperatures in the Late Cretaceous. The approximate erosional surface is indicated by the black dashed lines in the restored sections, and restored Cenozoic faults are indicated by gray dashed lines. RSCM and conodont alteration index (CAI) data are indicated by yellow and green diamonds, respectively.

Table S1. Parameters for conductive thermal curves. Range of values used in Monte-Carlo type simulations, controlled values for the moderate geotherm [50,51], and a range of values for the preferred fit.

Curve	Surface temperature (T_0) °C	Surface heat flux (q_s) mW m^{-2}	Mantle heat flux (q_m) mW m^{-2}	Radiogenic heating (ρ H_0) µW m^{-3}	Thermal conductivity (k) W/m/K	e-folding distance (h_r) (m)	Upper crust thermal gradient (°C/km)	Reference
**Monte-Carlo range**	0 – 20	Eq. 1	1 – 50	1 – 10	2 – 5	0 – Moho		1,2
**Control**	0	65	22	2.65	2.7	16.2×10^3	24*	1,2
**Preferred data fit**	10	100 – 125	2 – 14	5.5 – 10	2-2.3	11 – 16×10^3	40 – 50*	3

*Values approximated from the resultant curves.
References: (1) Vilà et al., (2010); (2) Turcotte and Schubert (2014); (3) This study

2. Discussion of heating mechanisms and possible slab refrigeration

Peak crustal temperatures may have been driven by combined processes of crustal thickening, radiogenic heating, fluid-flux melting, and dehydration of the subducting Farallon slab that metasomatized much of the North American lithosphere up to ~1000 km east of the subduction trench[15,52–57]. Regardless of the origin of extreme crustal heating, regional P-T-t estimates and argon thermochronology indicate peak thermal conditions were attained ca. 90 Ma and persisted until ca. 60 Ma[15]. Our working hypothesis is that the subducting flat slab hydrated the mantle lithosphere and lower crust to drive partial melting[57,58] that advected heat to the mid-upper crust, which is

consistent with the spatiotemporal onset of flat-slab subduction, peraluminous two-mica granite plutonism across the hinterland, and peak regional metamorphic conditions. A potential caveat is that it has been hypothesized that Laramide flat-slab subduction could have refrigerated the upper plate to cool the thermal structure of the overlying crust[59,60]. There would have been a competition between melt-induced heat advection and refrigeration from the colder oceanic slab, but our observations of an elevated thermal state in the upper-mid crust requires that heating dominated for most crustal levels, potentially due to a deep refrigerating slab or delayed cooling[60]. Conductive thermal modeling shows that cooling of the upper crust can take ca. 4 Myr to 32 Myr for slab depths of 50 km to 120 km, respectively[60]. That is, it may have taken 10s of Myr for the slab to refrigerate the thickened hinterland crust after subduction of the Conjugate Shatsky (90 – 80 Ma[61,62]) or the Conjugate Hess (ca. 75 Ma[63]) rises. Furthermore, the subducted oceanic plateaus may have had limited longitudinal extent, as shown via plate reconstructions[62], which complicates the impact of direct slab refrigeration versus slab-related hydration and melting of the upper plate.

3. Basal shear analytical solution shows lower-crust decoupling

To show how a hot and weak lower crust could decouple the upper-crust in the Cordillera hinterland from basal tractions from flat-slab subduction of the Farallon plate, we employ a simple Couette flow model for a two-layer fluid. This analytical solution shows the velocity distribution of two immiscible incompressible viscous fluids with different viscosities between two infinite, horizontal parallel plates. The upper plate is fixed, while the bottom plate is moving at a constant velocity of 50 mm/yr to similar basal drag from flat-slab subduction[64]. Further derivations of the Navier-Stokes equations in

ref. [51] solves for the velocity distribution in the lower u_1 and upper plate u_2, assuming u_1 = u_2 at their interface:

$$u_1 = \frac{\frac{U}{h}}{\frac{\mu_1}{\mu_2}+1} y + U - \frac{\frac{U}{h}}{\frac{\mu_1}{\mu_2}+1} 2h \tag{1a}$$

$$u_2 = \frac{\frac{U}{h}}{\frac{\mu_2}{\mu_1}+1} y \tag{1b}$$

where U is the lower plate velocity, h is the layer thickness, y is depth, and μ_1 and μ_2 are the viscosity of upper and lower layers, respectively. We assume the upper and lower crust are each ~30-km-thick (Fig. S2A). We model the resultant shear-strain in the upper crust for two scenarios: (i) one with a weak lower crust that matches our rheologic models for a hot Cordilleran hinterland, and (ii) one with a stronger lower crust that approximates a colder foreland region. The weak lower crust results in low shear strains in the upper crust (1×10^{-16} s^{-1}), whereas the stronger lower crust results in greater shear strains in the upper crust (1×10^{-14} s^{-1}) (Fig. S2B). This analytical solution shows that a hot, low-viscosity lower crust may promote lower crust mobility and decoupling of Laramide deformation.

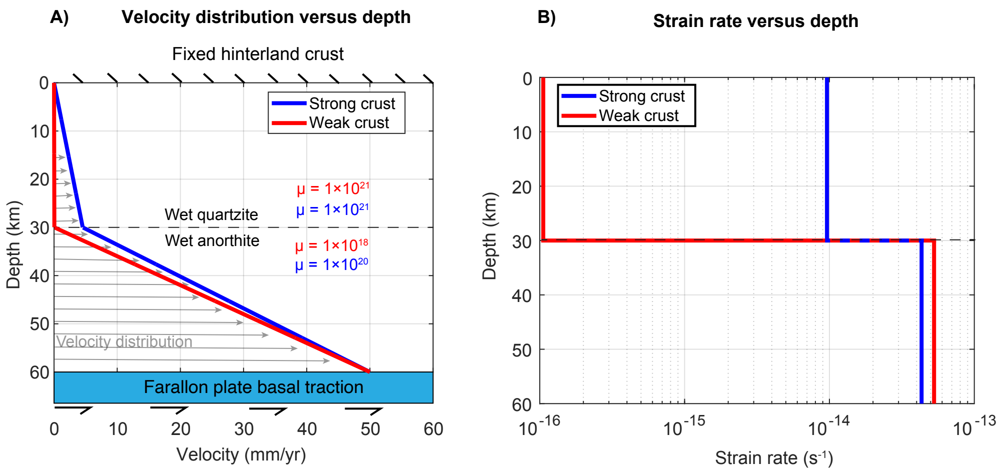

Fig. S2. Hinterland velocity and strain rate distribution. Velocity and strain rate distribution using a simple Couette flow model for a two-layer fluid. The upper plate is fixed, and the lower plate is moving at a constant velocity. (A) Velocity distribution of the hinterland crust using viscosity values derived from our modeled and cold geotherms. (B) Modeled strain rate of the upper and lower crust.

Supplementary References

1. Lee, J. K., Williams, I. S. & Ellis, D. J. Pb, U and Th diffusion in natural zircon. *Nature* **390**, 159–162 (1997).
2. Rubatto, D. Zircon trace element geochemistry: partitioning with garnet and the link between U–Pb ages and metamorphism. *Chemical geology* **184**, 123–138 (2002).
3. Parrish, R. R. U–Pb dating of monazite and its application to geological problems. *Canadian Journal of Earth Sciences* **27**, 1431–1450 (1990).
4. Spencer, K. *et al.* Campaign-style titanite U–Pb dating by laser-ablation ICP: Implications for crustal flow, phase transformations and titanite closure. *Chemical Geology* **341**, 84–101 (2013).
5. Scherer, E. E., Cameron, K. L. & Blichert-Toft, J. Lu–Hf garnet geochronology: closure temperature relative to the Sm–Nd system and the effects of trace mineral inclusions. *Geochimica et Cosmochimica Acta* **64**, 3413–3432 (2000).
6. Cheng, H., King, R., Nakamura, E., Vervoort, J. & Zhou, Z. Coupled Lu–Hf and Sm–Nd geochronology constrains garnet growth in ultra-high-pressure eclogites from the Dabie orogen. *Journal of metamorphic Geology* **26**, 741–758 (2008).
7. Tirone, M. *et al.* Rare earth diffusion kinetics in garnet: experimental studies and applications. *Geochimica et Cosmochimica Acta* **69**, 2385–2398 (2005).
8. Harrison, T. M., Célérier, J., Aikman, A. B., Hermann, J. & Heizler, M. T. Diffusion of 40Ar in muscovite. *Geochimica et Cosmochimica Acta* **73**, 1039–1051 (2009).
9. Harrison, T. M., Duncan, I. & Mcdougall, I. Diffusion of 40Ar in biotite: temperature, pressure and compositional effects. *Geochimica et Cosmochimica Acta* **49**, 2461–2468 (1985).
10. Dahl, P. S. The crystal-chemical basis for Ar retention in micas: inferences from interlayer partitioning and implications for geochronology. *Contributions to Mineralogy and Petrology* **123**, 22–39 (1996).
11. Hames, W. & Bowring, S. An empirical evaluation of the argon diffusion geometry in muscovite. *Earth and Planetary Science Letters* **124**, 161–169 (1994).
12. Zuza, A. V., Dee, S., Henry, C. D., Ressel, M. W. & Thorman, C. H. Geologic Map of the Independence Valley NW Quadrangle, Elko County, Nevada. (2019).
13. Levy, D. A. Timescales and mechanisms of crustal thickening and post-orogenic extension in the North American Cordillera. (2022).
14. Miller, E., Gans, P., Wright, J., Sutter, J. & Ernst, W. Metamorphic history of the east-central Basin and Range province: Tectonic setting and relationship to magmatism. *Metamorphism and crustal evolution, western conterminous United States, Rubey* **7**, 649–682 (1988).
15. Miller, E. L. & Gans, P. B. Cretaceous crustal structure and metamorphism in the hinterland of the Sevier thrust belt, western US Cordillera. *Geology* **17**, 59–62 (1989).
16. Wright, J. E. & Wooden, J. New Sr, Nd, and Pb isotopic data from plutons in the northern Great Basin: Implications for crustal structure and granite petrogenesis in the hinterland of the Sevier thrust belt. *Geology* **19**, 457–460 (1991).
17. Lee, S.-Y., Barnes, C. G., Snoke, A. W., Howard, K. A. & Frost, C. D. Petrogenesis of Mesozoic, peraluminous granites in the Lamoille Canyon area, Ruby Mountains, Nevada, USA. *Journal of Petrology* **44**, 713–732 (2003).
18. McGrew, A. J. & Snoke, A. W. SHRIMP-RG U-Pb isotopic systematics of zircon from the Angel Lake orthogneiss, East Humboldt Range, Nevada: Is this really Archean crust?: COMMENT. *Geosphere* **6**, 962–965 (2010).

19. Howard, K. A. *et al.* Episodic growth of a Late Cretaceous and Paleogene intrusive complex of pegmatitic leucogranite, Ruby Mountains core complex, Nevada, USA. *Geosphere* **7**, 1220–1248 (2011).
20. Wright, J. E. & Snoke, A. W. Tertiary magmatism and mylonitization in the Ruby-East Humboldt metamorphic core complex, northeastern Nevada: U-Pb geochronology and Sr, Nd, and Pb isotope geochemistry. *Geological Society of America Bulletin* **105**, 935–952 (1993).
21. Snoke, A., McKee, E. & Stern, T. Plutonic, metamorphic, and structural chronology in the northern Ruby Mountains. *Nevada: A preliminary report: Geological Society of America Abstracts with Programs* **11**, 520–521 (1979).
22. McGrew, A. J., Peters, M. T. & Wright, J. E. Thermobarometric constraints on the tectonothermal evolution of the East Humboldt Range metamorphic core complex, Nevada. *Geological Society of America Bulletin* **112**, 45–60 (2000).
23. Hallett, B. W. *Metamorphic history and timescales of cooling and exhumation of partially melted rocks: Studies in the North American Cordillera*. (Rensselaer Polytechnic Institute, 2012).
24. Premo, W. R., Castiñeiras, P. & Wooden, J. L. SHRIMP-RG U-Pb isotopic systematics of zircon from the Angel Lake orthogneiss, East Humboldt Range, Nevada: Is this really Archean crust? *Geosphere* **4**, 963–975 (2008).
25. Premo, W. R., Castiñeiras, P. & Wooden, J. L. SHRIMP-RG U-Pb isotopic systematics of zircon from the Angel Lake orthogneiss, East Humboldt Range, Nevada: Is this really Archean crust? REPLY. *Geosphere* **6**, 966–972 (2010).
26. Premo, W. R., Moscati, R., McGrew, A. & Snoke, A. New U-Pb zircon geochronology of Precambrian paragneisses and late Phanerozoic orthogneisses of the Angel Lake–Lizzies Basin region of the East Humboldt Range, northeastern Nevada: A comparison with the thermal chronology at Lamoille Canyon in the adjacent Ruby Mountains. in vol. 46 33 (2014).
27. Wills, M. A. A metamorphic pressure-temperature time path from the Wood Hills, Elko County, eastern Nevada. (2014).
28. Camilleri, P. A. & Chamberlain, K. R. Mesozoic tectonics and metamorphism in the Pequop Mountains and Wood Hills region, northeast Nevada: Implications for the architecture and evolution of the Sevier orogen. *Geological Society of America Bulletin* **109**, 74–94 (1997).
29. Blackford, N. R. *et al.* Late Cretaceous upper-crustal thermal structure of the Sevier hinterland: Implications for the geodynamics of the Nevadaplano. *Geosphere* **18**, 183–210 (2022).
30. Lee, D., Stacey, J., Fischer, L., Peterman, Z. & Schnabel, D. Muscovite phenocrystic two mica granites of northeastern Nevada are Late Cretaceous in age. *US Geological Survey Bulletin* **1622**, 31–39 (1986).
31. Lee, D. E., Marvin, R. F., Stern, T. & Peterman, Z. E. Modification of potassium-argon ages by Tertiary thrusting in the Snake Range, White Pine County, Nevada. *Geological Survey research* D92–D102 (1970).
32. Lee, D. E., Marvin, R. F. & Mehnert, H. H. *A radiometric age study of Mesozoic-Cenozoic metamorphism in eastern White Pine County, Nevada, and nearby Utah*. (US Government Printing Office, 1980).
33. Lee, D. & Fischer, L. Cretaceous metamorphism in the northern Snake Range, Nevada, a metamorphic core complex. *Isochron/West* **42**, 3–7 (1985).
34. Cooper, F. J., Platt, J. P., Anczkiewicz, R. & Whitehouse, M. Footwall dip of a core complex detachment fault: Thermobarometric constraints from the northern Snake Range (Basin and Range, USA). *Journal of Metamorphic Geology* **28**, 997–1020 (2010).

35. Gottlieb, E. S. Geologic Insights from Zircon Inheritance. (2017).
36. Rodgers, D. W. *Thermal and structural evolution of the southern Deep Creek Range, west central Utah and east central Nevada*. (Stanford University, 1987).
37. Lee, D. E., Stern, T. & Marvin, R. Uranium-thorium-lead isotopic ages for metamorphic monazite from the northern Snake Range, Nevada. *Isochron/west* **1**, 1–35 (1981).
38. Lee, J., Miller, E. L. & Sutter, J. F. Ductile strain and metamorphism in an extensional tectonic setting: A case study from the northern Snake Range, Nevada, USA. *Geological Society, London, Special Publications* **28**, 267–298 (1987).
39. Ketner, K. B. *Geologic maps showing structural modes in the Goshute Mountains and Toano Range, Elko County, Nevada*. (1997).
40. Dee, S., Henry, C. D., Ressel, M. W. & Zuza, A. V. Preliminary geologic map of the north half of the Independence Valley NW quadrangle and the adjacent part of the Independence Valley NE quadrangle, Elko County, Nevada. *Nevada Bureau of Mines and Geology Open-File Report 17-6, scale 1: 24,000* 4 (2017).
41. Hope, R. A. *Geologic map of the Spruce Mountain quadrangle, Elko County, Nevada*. (1972).
42. Pape, J. R., Seedorff, E., Baril, T. C. & Thompson, T. B. Structural reconstruction and age of an extensionally faulted porphyry molybdenum system at Spruce Mountain, Elko County, Nevada. *Geosphere* **12**, 237–263 (2016).
43. Colgan, J. P., Howard, K. A., Fleck, R. J. & Wooden, J. L. Rapid middle Miocene extension and unroofing of the southern Ruby Mountains, Nevada. *Tectonics* **29**, (2010).
44. Coats, R. R. *Geology of Elko County, Nevada*. vol. 101 (Nevada Bureau of Mines and Geology, University of Nevada-Reno, 1987).
45. Stewart, J. H. Geology of Nevada. *Nevada Bureau of Mines and Geology special publication* **4**, 136 (1980).
46. Henry, C. & Thorman, C. Preliminary geologic map of the Pequop Summit quadrangle. *Elko County, Nevada: Nevada Bureau of Mines and Geology Open-File Report* 15–8 (2015).
47. Zuza, A. V., Henry, C. D., Dee, S., Thorman, C. H. & Heizler, M. T. Jurassic–Cenozoic tectonics of the Pequop Mountains, NE Nevada, in the North American Cordillera hinterland. *Geosphere* **17**, 2078–2122 (2021).
48. Konstantinou, A., Strickland, A., Miller, E. L. & Wooden, J. P. Multistage Cenozoic extension of the Albion–Raft River–Grouse Creek metamorphic core complex: Geochronologic and stratigraphic constraints. *Geosphere* **8**, 1429–1466 (2012).
49. Long, S. P. Magnitudes and spatial patterns of erosional exhumation in the Sevier hinterland, eastern Nevada and western Utah, USA: Insights from a Paleogene paleogeologic map. *Geosphere* **8**, 881–901 (2012).
50. Vilà, M., Fernández, M. & Jiménez-Munt, I. Radiogenic heat production variability of some common lithological groups and its significance to lithospheric thermal modeling. *Tectonophysics* **490**, 152–164 (2010).
51. Turcotte, D. & Schubert, G. *Geodynamics*. (Cambridge University Press, 2014). doi:10.1017/CBO9780511843877.
52. Douce, P., Humphreys, E. D. & Johnston, A. D. Anatexis and metamorphism in tectonically thickened continental crust exemplified by the Sevier hinterland, western North America. *Earth and Planetary Science Letters* **97**, 290–315 (1990).
53. Humphreys, E. *et al.* How Laramide-age hydration of North American lithosphere by the Farallon slab controlled subsequent activity in the western United States. *International Geology Review* **45**, 575–595 (2003).

54. Lee, C.-T. A. Trace element evidence for hydrous metasomatism at the base of the North American lithosphere and possible association with Laramide low-angle subduction. *The Journal of Geology* **113**, 673–685 (2005).
55. Smith, D. Antigorite peridotite, metaserpentinite, and other inclusions within diatremes on the Colorado Plateau, SW USA: implications for the mantle wedge during low-angle subduction. *Journal of Petrology* **51**, 1355–1379 (2010).
56. Chapman, J. B. *et al.* The north American cordilleran anatectic belt. *Earth-Science Reviews* **215**, 103576 (2021).
57. Segee-Wright, G. *et al.* Halogen enrichment in the North American lithospheric mantle from the dehydration of the Farallon plate. *Geochimica et Cosmochimica Acta* **348**, 187–205 (2023).
58. Jödicke, H. *et al.* Fluid release from the subducted Cocos plate and partial melting of the crust deduced from magnetotelluric studies in southern Mexico: Implications for the generation of volcanism and subduction dynamics. *Journal of Geophysical Research: Solid Earth* **111**, (2006).
59. Dumitru, T. A., Gans, P. B., Foster, D. A. & Miller, E. L. Refrigeration of the western Cordilleran lithosphere during Laramide shallow-angle subduction. *Geology* **19**, 1145–1148 (1991).
60. Liu, X., Currie, C. A. & Wagner, L. S. Cooling of the continental plate during flat-slab subduction. *Geosphere* **18**, 49–68 (2022).
61. Liu, L. *et al.* The role of oceanic plateau subduction in the Laramide orogeny. *Nature Geoscience* **3**, 353–357 (2010).
62. Axen, G. J., van Wijk, J. W. & Currie, C. A. Basal continental mantle lithosphere displaced by flat-slab subduction. *Nature Geoscience* **11**, 961–964 (2018).
63. Schwartz, J. J. *et al.* Magmatic surge requires two-stage model for the Laramide orogeny. *Nature Communications* **14**, 3841 (2023).
64. Yonkee, W. A. & Weil, A. B. Tectonic evolution of the Sevier and Laramide belts within the North American Cordillera orogenic system. *Earth-Science Reviews* **150**, 531–593 (2015).
