# Supplementary material for: Hot Cordilleran hinterland promoted lower crust mobility and decoupling of Laramide deformation
Source: Nat Commun. 2024 May 4;15:3750. doi: 10.1038/s41467-024-48182-8 (PMC11069518; doi:10.1038/s41467-024-48182-8)
Supplement: Supplementary file 3 — Description of Additional Supplementary Files [file 41467_2024_48182_MOESM3_ESM.pdf]

Description of Additional Supplementary Files

Supplementary Dataset 1. Compilation of temperature versus depth data across the Cordilleran hinterland.

Supplementary Dataset 2. IFORS raw data from this study. IFORS can be downloaded here: http://www.sediment.uni-goettingen.de/download/.

Supplementary Dataset 3. Additional remarks for the southern Ruby Mountains.
